# HERC6 Is the Main E3 Ligase for Global ISG15 Conjugation in Mouse Cells

**DOI:** 10.1371/journal.pone.0029870

**Published:** 2012-01-17

**Authors:** Diede Oudshoorn, Sander van Boheemen, Maria Teresa Sánchez-Aparicio, Ricardo Rajsbaum, Adolfo García-Sastre, Gijs A. Versteeg

**Affiliations:** 1 Department of Microbiology, Mount Sinai School of Medicine, New York, New York, United States of America; 2 Department of Medicine, Mount Sinai School of Medicine, New York, New York, United States of America; 3 Global Health and Emerging Pathogens Institute, Mount Sinai School of Medicine, New York, New York, United States of America; Kantonal Hospital St. Gallen, Switzerland

## Abstract

Type I interferon (IFN) stimulates expression and conjugation of the ubiquitin-like modifier IFN-stimulated gene 15 (ISG15), thereby restricting replication of a wide variety of viruses. Conjugation of ISG15 is critical for its antiviral activity in mice. HECT domain and RCC1-like domain containing protein 5 (HerC5) mediates global ISGylation in human cells, whereas its closest relative, HerC6, does not. So far, the requirement of HerC5 for ISG15-mediated antiviral activity has remained unclear. One of the main obstacles to address this issue has been that no HerC5 homologue exists in mice, hampering the generation of a good knock-out model. However, mice do express a homologue of HerC6 that, in contrast to human HerC6, can mediate ISGylation.

Here we report that the mouse HerC6 N-terminal RCC1-like domain (RLD) allows ISG15 conjugation when replacing the corresponding domain in the human HerC6 homologue. In addition, sequences in the C-terminal HECT domain of mouse HerC6 also appear to facilitate efficient ISGylation. Mouse HerC6 paralleled human HerC5 in localization and IFN-inducibility. Moreover, HerC6 knock-down in mouse cells abolished global ISGylation, whereas its over expression enhanced the IFNβ promoter and conferred antiviral activity against vesicular stomatitis virus and Newcastle disease virus. Together these data indicate that HerC6 is likely the functional counterpart of human HerC5 in mouse cells, suggesting that HerC6^−/−^ mice may provide a feasible model to study the role of human HerC5 in antiviral responses.

## Introduction

Type I interferons (IFN) are induced in response to infection and mediate the expression of antiviral IFN-stimulated genes (ISGs) [1]. ISG15 is an 15 kDa ubiquitin-like modifier consisting of two ubiquitin-like domains that is early and abundantly induced by type I IFN [2]. Similar to ubiquitin, ISG15 is conjugated to lysines on numerous target proteins through the action of specific E1-activating, E2-conjugating, and E3-ligase enzymes, all of which are also IFN-inducible [3], [4], [5], [6]. Previous knock-down studies demonstrated that HerC5 is the main ISG15 E3 ligase to mediate global conjugation in human cells, whereas HerC6 -its closest relative- was devoid of any ISG15 conjugating activity [3], [7].

The HerC protein family consists of six members, two of which (HerC1 and HerC2) are substantially larger than the others (HerC3–6) [8], [9]. They share a C-terminal HECT ubiquitin E3-ligase domain and an N-terminal RCC1-like domain (RLD). The exact function of the RLDs in these proteins has remained elusive [10]. Phylogenetic analysis of the HerC protein family indicated that HerC5 is only present in higher vertebrates [9].

So far, ISG15-mediated antiviral activity has been shown most convincingly *in vivo* by means of infections in ISG15^−/−^ mice [11], [12]. In addition, overexpression of ISG15 by a recombinant Sindbis virus results in *in vivo* attenuation. Interestingly, recombinant Sindbis viruses overexpressing unconjugatable ISG15 mutants were not attenuated, suggesting that attachment of mouse ISG15 to lysines of target proteins is critical for antiviral function [11]. A recent report indicated that HerC5 may globally target *de novo* synthesized proteins for ISG15 conjugation, thereby making viral proteins major targets for ISGylation [13]. However, the question remains if HerC5 and global ISGylation are important for antiviral activity *in vivo* as ISGylation-mediated antiviral effects might be due to other minor ISG15 E3 ligases with more narrow specificity, such as EFP [14].

Development of a mouse model to address this question has been hampered by the fact that mice do not possess a HerC5 homologue. The closest mouse gene to human HerC5 is the mouse homologue of human HerC6 [9]. Since human HerC6 does not exert any ISG15 conjugating activity, the question remains what the functional counterpart of human HerC5 is in mice.

Recently, we reported that mouse HerC6, but not human HerC6 could mediate ISG15 conjugation onto cellular proteins in a transfection system [7]. Here we set out to establish if HerC6 in mice functionally parallels human HerC5 and report that HerC6 acts as the main ISG15 E3 ligase to mediate global ISG15 conjugation in mouse cells.

## Results

### The mouse HerC6 RLD confers efficient ISG15 conjugation to human HerC6

**Figure 1:**
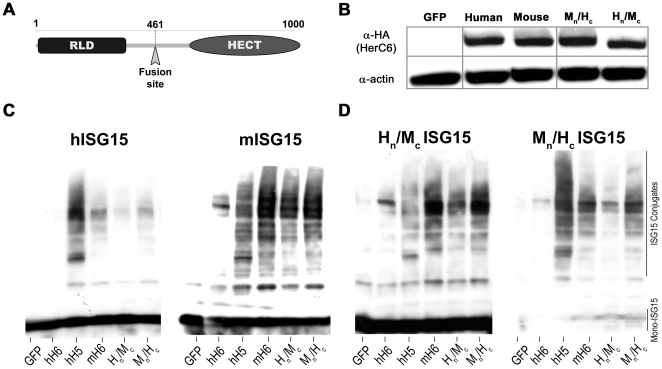
The mouse HerC6 RLD confers efficient ISG15 conjugation. A. Schematic overview of the HerC6 chimeras with the RLD and HECT domains. B. HerC expression levels were determined in HEK-293T cells transfected with plasmids expressing wild-type HA-tagged HerC proteins and human-mouse chimeric HerC6 proteins. C/D. HEK-293T cells transfected with plasmids expressing human E1 and E2 enzymes, indicated HerC proteins and either V5-tagged human or mouse wild-type ISG15 (C) or human-mouse ISG15 chimeras (D) were analyzed for global ISGylation by V5-specific immunoblot.

In order to assess what moiety of the mouse HerC6 protein confers ISG15 E3 ligase activity in comparison to human HerC6, which doesn't possess ISG15 E3 activity, we constructed plasmids expressing chimeric human/mouse HerC6 proteins. Either the N-terminal RLD or the C-terminal HECT domain was exchanged with the equivalent of the alternate species (Fig. 1a). Analysis of the chimeric proteins by Western blot demonstrated that the chimeras express to similar levels as their wild-type equivalents (Fig. 1b).

Next, these constructs were tested for their ability to conjugate ISG15 to cellular targets in the presence of over-expressed E1 and E2 enzymes. As previously described, both hHerC5 and mHerC6 effectively conjugated both human and mouse ISG15, whereas no ISG15 conjugates were detected in cells overexpressing hHerC6 (Fig. 1c, lanes hH6, hH5 and mH6). Replacement of the human HerC6 N-terminal RLD by its mouse equivalent almost completely restored ISGylation (Fig. 1c, compare lanes mH6 and M_N_H_C_), suggesting that mainly differences in the RLD between hHerC6 and mHerC6 allow mHerC6 to conjugate ISG15. However, exchange of the mouse HerC6 C-terminus partially restored ISGylation by hHerC6 (Fig. 1c, compare lanes hH6 and H_N_M_C_), indicating that the ability of mHerC6 to function as an E3 ligase compared to hHerC6 also partially relies on differences in the protein's C-terminal half that contains the HECT domain. The same constructs were analyzed in the presence of the mouse E1, E2 and ISG15. Despite the fact that mouse ISG15 was much more efficiently conjugated in the presence of its native conjugation machinery, similar results were obtained with the HerC6 chimeras (data not shown).

The results described in figure 1c suggested that mainly the mHerC6 N-terminal half is required for efficient ISG15 conjugation, irrespective of the species origin of ISG15. Conjugation of ISG15 chimeric proteins harboring either the N- or C-terminal Ub-like domain of mouse or human ISG15 confirmed and strengthened this observation. The mHerC6 N-terminal half almost completely restored E3 activity in hHerC6 for both chimeric ISG15 proteins (Fig. 1d, compare lanes mH6 and M_N_H_C_), although the mHerC6 C-terminal half also partially restored ISG15 conjugation (Fig. 1d, compare lanes hH6 and H_N_M_C_). Moreover, the mISG15 and H_N_M_C_ ISG15 molecules were consistently conjugated with higher efficiency than hISG15 or the M_N_H_C_ ISG15 chimera. Together with the observation that conjugation of endogenous ISG15 upon IFN treatment is consistently more robust in mouse cells than in human cells (data not shown), these latter data seem to indicate that mISG15 may be more efficiently conjugated than hISG15 and that this property seems to reside in its C-terminal Ub-like domain (Fig. 1., compare panel c and d).

In conclusion, these data demonstrate that mouse HerC6 has ISG15 E3 ligase activity and that this activity mainly depends on features in the N-terminal half of the protein compared to human HerC6. Next we investigated if expression of endogenous mHerC6 is also stimulated by type I IFN and localizes similar to human HerC5 and HerC6.

### mHerC6 is induced by type I interferon and localizes exclusively in the cytoplasm

**Figure 2:**
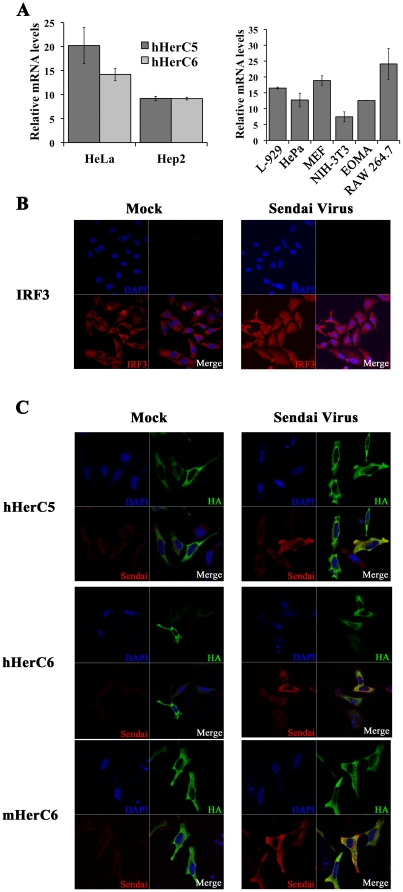
mHerC6 is induced by type I interferon and localizes exclusively in the cytoplasm. A. Indicated murine and human cell lines were stimulated with recombinant type I IFN and subsequently analyzed for HerC mRNA regulation by RT-qPCR. Values are relative fold-change over mock-induced samples. Experiments were reproduced at least twice; a representative experiment is shown. Error bars represent standard deviation of qPCR replicates. B. HeLa cells transfected with an empty control plasmid and subsequently infected with SeV were immuno-stained with IRF-3 specific antibodies. C. Localization of overexpressed HA-tagged HerC proteins in the presence of subsequent SeV infection was determined in HeLa cells by immuno-fluorescence assay using HA- and SeV-specific antibodies.

Although human HerC5 and HerC6 differ in their ability to conjugate ISG15, they share their inducibility by type I IFN and their localization in the cytoplasm [9]. We investigated if mouse HerC6 is also IFN-inducible and localizes to a sub-cellular site similar to its human relatives. To this end, HerC5 and hHerC6 mRNA upregulation was measured in human Hep2 and HeLa cells stimulated with IFNβ and compared to mHerC6 mRNA regulation in several IFN-stimulated mouse cell lines (Fig. 2a).

IFN stimulation upregulated human HerC5 and HerC6 mRNA in both human cell lines, albeit to a lesser extent in Hep2 than in HeLa cells (Fig. 2a). HerC5 and HerC6 were both upregulated 9-fold in Hep2 cells, whereas HerC5 mRNA increased slightly more (20-fold) compared to HerC6 mRNA (14-fold) in HeLa cells (Fig. 2a, left panel). The same amount of IFN induced an average 12-fold upregulation of mHerC6 mRNA levels in the six mouse cell lines tested (Fig. 2a, right panel), varying from 7-fold in NIH-3T3 cells to 25-fold in the macrophage cell line RAW 264.7.

Subsequently, localization of HerC proteins was examined by immuno-fluorescence assays of HeLa cells transfected with HerC5/6 expression plasmids. Since all tested HerC5/6 proteins are IFN-inducible, we investigated sub-cellular localization both under non-induced conditions, as well as during infection with SeV, a potent inducer of the type I IFN system [14].

Infection with SeV translocated IFN-regulatory factor 3 (IRF-3), a transcription factor critical for efficient IFNβ induction [14], to the nucleus (Fig. 2b). These results indicated that the assay conditions were correct for detecting sub-cellular changes of molecules important for the IFN-response. In agreement with previously published observations, human HerC5 and HerC6 localized exclusively to the cytoplasm in mock-induced cells (Fig. 2c). Despite the nuclear translocation of IRF-3, which is considered a hallmark of productive IFN induction and hence SeV infection, human HerC5 and HerC6 localization was unchanged during SeV infection (Fig. 2c). Similarly, mouse HerC6 localized in the cytoplasm and its localization remained unchanged during SeV infection (Fig. 2c). Together these data show that mouse HerC6 IFN-inducibility and localization in mouse cells are very similar to that of hHerC5/6 in human cells.

### mHerC6 is essential for global ISG15 conjugation in mouse cells

Since mouse HerC6 was able to facilitate ISG15 conjugation in an overexpression system, we next set out to determine if HerC6 is the main E3 ligase mediating global ISGylation in mouse cells. To that end mouse L-929 cells were transfected with siRNAs specifically targeting mHerC6. As a control siRNAs targeting hHerC6 (but not mHerC6) were used.

**Figure 3:**
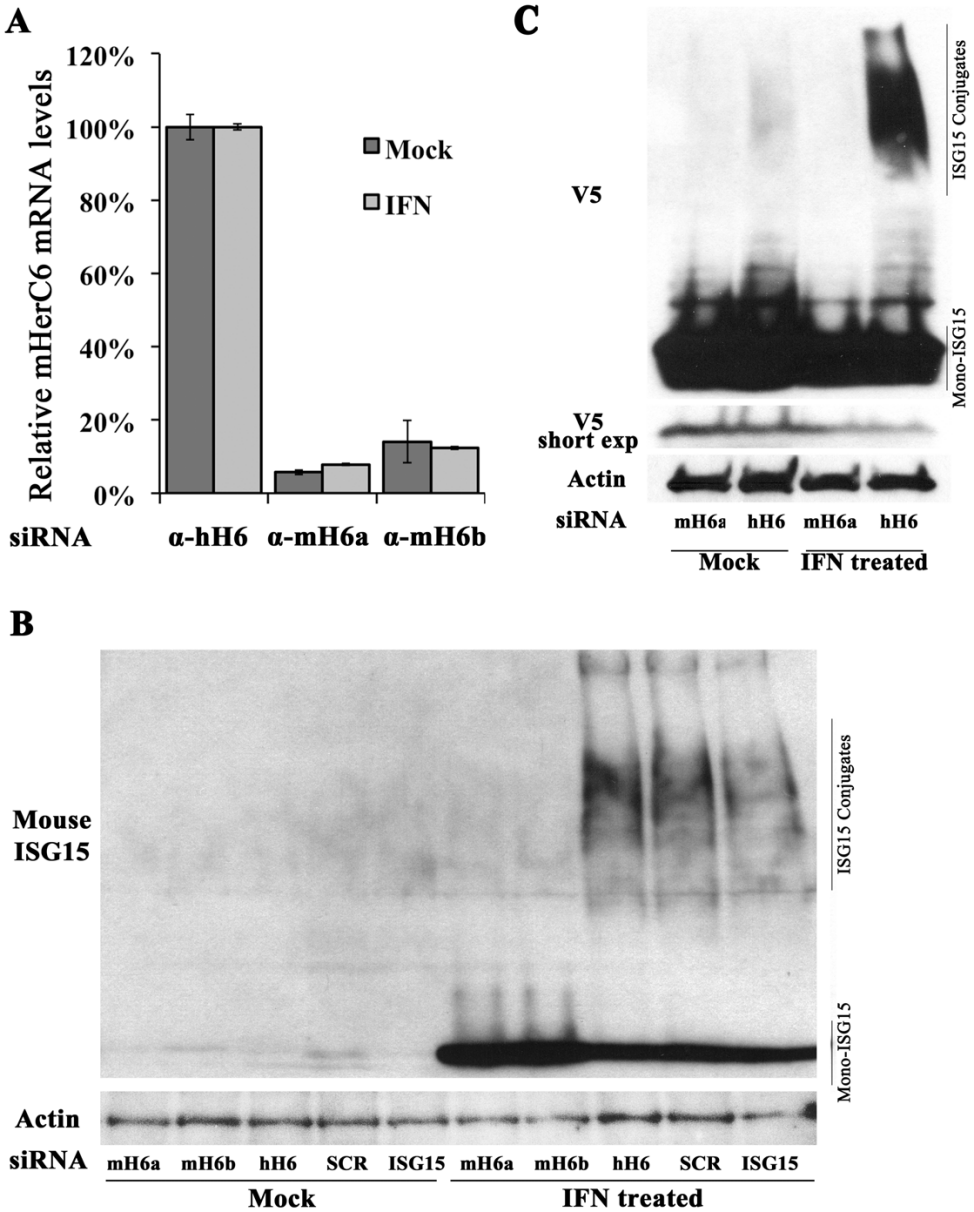
mHerC6 is essential for global cellular ISG15 conjugation in mouse cells. (A) mRNA knock-down of HerC6 was analyzed by RT-qPCR in mouse L929 cells transfected with siRNAs specifically targeting human or mouse HerC6 and subsequently treated with recombinant type I IFN. mRNA levels in mHerC6 knock-down cells are plotted relative to mRNA levels in cells with non-targeting hHerC6 siRNA. Experiments were reproduced at least twice; a representative experiment is shown. Error bars represent standard deviation of qPCR replicates. B/C. L-929 cells were transfected with (B) indicated siRNAs, stimulated with IFN for 48 h and probed for endogenous ISG15 on a Western blot or (C) simultaneously transfected with a V5-tagged mouse ISG15 plasmid and indicated siRNAs, stimulated with IFN for 48 h and subsequently analyzed for global ISG15 conjugation by V5-specific immunoblot.

Firstly, RNA was extracted from siRNA transfected cells and specific knock-down was determined by real-time RT-PCR with species-specific HerC6 primers. We compared IFN treated samples to mock samples to assure that IFN induction would not abolish knock-down. Efficiency of knock-down was determined by normalizing the observed knock-down to the human specific siRNA controls. Four different commercial siRNAs targeting mHerC6 were tested. Efficient and specific knock-down was observed for all of them, whereas controls had no effect on mHerC6 mRNA levels (data not shown). The assay was repeated with the two most efficient siRNAs (mHerC6a and –b) which specifically reduced mHerC6 mRNA in mouse cells by >85% compared to the control (Fig. 3a).

Finally, global ISGylation was analyzed in the context of mHerC6 knockdown. We hypothesized that if mHerC6 were the functional equivalent of human HerC5, knock-down of mHerC6 in L-929 would abolish global ISGylation. We transfected siRNAs targeting either mHerC6, hHerC6, mouse ISG15 or a scrambled control in L-929 cells and analyzed ISGylation 48 h after IFN stimulation. ISG15 expression was only upregulated in IFN-treated cells. Moreover, ISGylation was detected when a non-targeting control siRNA was transfected, whereas ISGylation was specifically abolished by knockdown of mHerC6, indicating that mHerC6 is essential for global ISGylation in mouse cells. Two different mHerC6-specific siRNAs demonstrated the same effect (Fig. 3b), thus making off-target effects by the siRNAs unlikely. The increase of unconjugated ISG15 upon knockdown of mHerC6 is likely due to accumulation of ISG15 that would otherwise be conjugated.

To validate these results in a transfection system in which ISG15 expression is controlled, we subsequently co-transfected a V5-tagged ISG15 expression plasmid together with a mHerC6-specific or control siRNA and stimulated the cells with IFN. ISG15 was expressed from a plasmid and thus at a constant level in all samples (Fig. 3c). However, global ISGylation was only detected upon IFN treatment (Fig. 3c), indicating that upregulation of the endogenous E1, E2 and E3 enzymes was IFN-dependent as expected. Moreover, transfection of four different siRNAs targeting mouse HerC6 abolished global ISGylation in comparison with the control siRNA (Fig. 3c, IFN treated lanes and Fig. S1). Taken together these data confirm that HerC6 is the main ISG15 E3-ligase in mouse cells and in that respect functionally parallels human HerC5, even though genetically it is more closely related to hHerC6. The use of four different mHerC6-specific siRNAs strongly demonstrated that the loss of global ISGylation upon mHerC6 knock-down is specific and not resulting from off-target effects.

### mHerC6 enhances IFNβ promoter induction and confers antiviral activity in mouse cells

**Figure 4:**
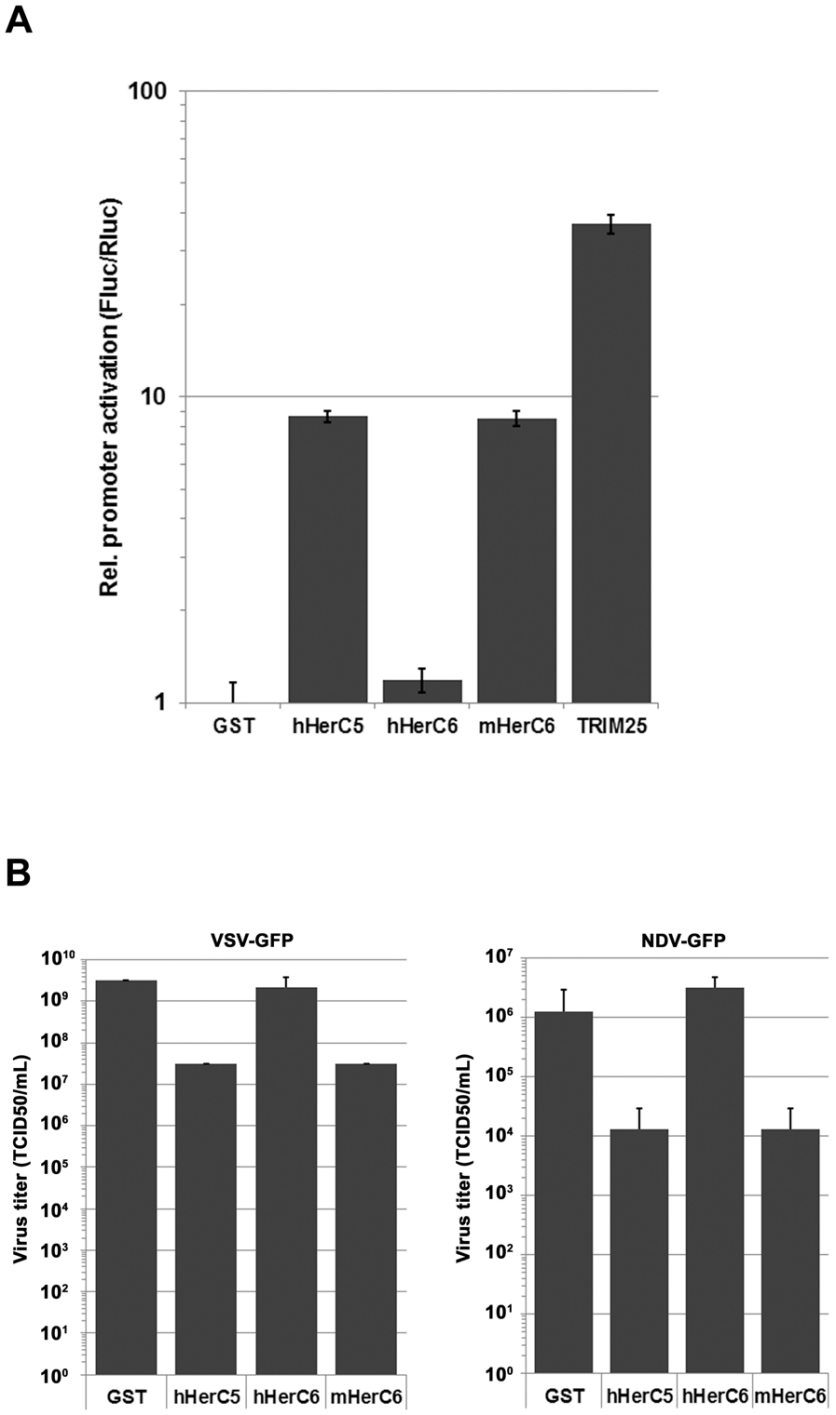
mHerC6 stimulates the IFNβ promoter and confers antiviral activity against VSV and NDV. (A) HEK-293T cells were transfected with an IFNβ reporter construct controlling firefly luciferase, a constitutively active renilla luciferase internal control plasmid, a limiting amount of a plasmid expressing a constitutively active form of RIG-I (RIG-I(2CARD)) and the indicated HerC5/6 proteins. After 24 h, cells were lysed and luciferase measured. Values were normalized to the internal control and plotted relative to the GST control. (B) L-929 cells were transfected with the indicated plasmids. After 48 h, the cells were infected with VSV-GFP or NDV-GFP at an m.o.i. of 5. At 7 h p.i. supernatant was harvested from the VSV-GFP infected cells and at 16 h p.i. from the NDV-GFP infected cells. The supernatants were tittered by TCID50 on HEK-293T cells.

Previous studies have demonstrated that hHerC5 positively regulates the IFNβ promoter and confers antiviral activity [15]. To investigate if the HerC5/6 proteins could enhance an innate immune signal initiated by the RNA sensor RIG-I, HEK-293T cells were transfected with an IFNβ reporter construct controlling firefly luciferase, a constitutively active renilla luciferase internal control plasmid, a limiting amount of a plasmid expressing a constitutively active form of RIG-I (RIG-I(2CARD)) and the indicated HerC5/6 proteins. TRIM25, a well-established activator of RIG-I, was used as a positive control [16]. As previously reported, hHerC5 enhanced IFNβ promoter activity (by 8.7 fold; Fig. 4a) over the unspecific control protein GST, whereas its hHerC6 relative without ISG15 E3 ligase activity did not further enhance promoter activity. In contrast, mHerC6 also further enhanced IFNβ activity by 8.5 fold, similar to its hHerC5 counterpart (Fig. 4a).

To address the antiviral properties of mHerC6 in mouse cells, we determined if HerC5/6 expression would confer antiviral resistance to VSV-GFP and NDV-GFP infection. We transfected L-929 cells with the indicated constructs and subsequently infected the cells with either vesicular stomatitis virus (VSV) or Newcastle disease virus (NDV) expressing GFP, and analyzed the produced virus secreted into the supernatant by TCID50 assay. In correlation with what has been previously reported, hHerC5 significantly reduced both VSV and NDV virus production by 100-fold, whereas its hHerC6 relative without E3 activity did not (Fig. 4b). Moreover, mHerC6 also reduced the titers of both viruses, highly similar to its human counterpart, hHerC5.

Taken together, these results indicate that mHerC6 parallels hHerC5 in its ability to enhance the IFNβ promoter and confer antiviral activity. In contrast, the hHerC6 protein did not have these activities, which correlates with its lack of E3 ligase activity. Moreover, the fact that the hHerC5 and mHerC6 proteins had activity in both human and mouse cells, is in line with our previous observation that hHerC5 and mHerC6 can mediate global ISGylation using ISG15 and the conjugation machinery of the opposite species.

## Discussion

Here we demonstrate that mouse HerC6 parallels human HerC5 in its localization, induction by type I IFN, antiviral activity and its role as main ISG15 E3 ligase in mouse cells. Together these findings indicate that humans and mice developed different HerC proteins to facilitate global ISG15 conjugation during evolution. The results from the silencing studies strongly suggest that both humans and mice only express a single protein to globally attach ISG15 to a wide range of target proteins during IFN induction (Fig. 3b and c). The fact that only higher vertebrates express both HerC5 and an IFN-induced human HerC6-like protein without global ISGylation activity, suggests that HerC5 may have arisen as an evolutionary response against pathogens specific to higher vertebrates, taking over the role of global ISGylation that HerC6 has in lower vertebrates.

No distinct function has been attributed to hHerC6 so far. Although it is clearly not essential for global ISGylation, we cannot rule out that it may possess E3 ligase activity for a more select range of target proteins or that it conjugates Ub-like modifiers different from ISG15. The results obtained with the human-mouse HerC6 chimeras show that the enzymatic HECT domain of human HerC6 is capable of ISG15 conjugation in the presence of the mouse HerC6 N-terminus (Fig. 1c). Therefore, a function of hHerC6 in the regulation of antiviral effects of ISG15 as an E3-ligase cannot be completely excluded, although in that case its N-terminal RLD would probably target a more selective set of proteins. It should be noted that the predicted binding site of the E2 enzyme also resides in the C-terminal half of HerC proteins, just upstream of the HECT domain. Thus, some of the increased ISGylation in chimeras containing the human HerC6 N-terminus and the mouse HerC6 C-terminus may indicate that mice and humans could use E2 enzymes that may be differentially compatible with global ISGylation.

We demonstrate that the previously reported IFNβ-inducing and antiviral effect exerted by hHerC5 [15], is shared by HerC6 in mouse cells (Fig. 4). Yet, hHerC6, which does not possess E3 ligase activity, failed to reduce VSV and NDV production or stimulate an IFNβ reporter. Shi *et. al.* also described antiviral effects of hHerC5 expression on SeV [15], which could potentially have lowered the virus levels in our localization studies (Fig. 2). Nevertheless, we demonstrate strong SeV antigen expression by immuno-fluorescence, which is highly similar in all infected samples, suggesting potentially only a minor effect by SeV reduction in our assay (Fig. 2).

The fact that the main global E3-ligases differ between mice and humans raises the question whether there are more differences in the ISG15 antiviral system. Conjugation of endogenous ISG15 and experiments with the ISG15 chimeras indicate that global conjugation of mISG15 under the conditions tested is more efficient than that of hISG15 and depends on its C-terminal Ub-like domain (Fig. 1c and 1d). Together with the observation that humans and mice adopted different HerC proteins during evolution for the same function of global ISGylation, this could indicate that other –hitherto unidentified- functional differences between human and mouse ISG15 exist.

A recent report indicated that HerC5 mainly conjugates ISG15 to newly synthesized proteins in tissue culture and may by that mechanism largely target *de novo* synthesized viral proteins during infection [13]. Moreover, additional studies have shown that conjugation of ISG15 is critical for antiviral function in mice [11]. However, it remains unclear if global ISGylation by HerC proteins is important to confer antiviral protection. Alternatively, conjugation by more specific E3 ligases may play an important role in antiviral restriction.

The findings in tissue culture put forward in this report indicate that targeted gene disruption of HerC6 will likely prove a reasonable model to study the role of global ISG15 E3 ligase activity in ISGylation-mediated antiviral activity *in vivo*.

## Materials and Methods

### Cells, viruses and IFN-β stimulation

HEK-293T, HeLa, Hep2, EOMA, MEF, NIH-3T3, Hepa and L-929 cells were acquired from the ATCC and maintained in Dulbecco's Modified Eagle's Medium (DMEM) supplemented with 10% Fetal Bovine Serum (FBS) and 100 IU/mL penicillin-streptomycin. Sendai virus Cantell strain was purchased from ATCC, grown for two days on 10-day old embryonated chicken eggs and tittered using HA assays with turkey red blood cells (Lampire Biological Laboratories, Cat# 7209403). Newcastle disease virus (NDV) expressing green fluorescent protein (GFP) (NDV-GFP) was grown in 10-day-old embryonated eggs (Charles River) and used for infections in the presence of TPCK trypsin [17]. Vesicular stomatitis virus (VSV) expressing GFP (VSV-GFP) was grown in BHK-21 cells [18].

### Cloning, plasmids and siRNAs

Expression plasmids for human Ube1L, UbcH8, HerC5, HerC6, ISG15 and mouse Ube1L, UbcM8, HerC6, ISG15, as well as plasmids expressing chimeric human/mouse and mouse/human ISG15 have been described previously [7]. The reporter plasmid expressing firefly luciferase under the IFNβ promoter was described previously [14]. Expression plasmid for flag-tagged RIG-I(2CARD) was previously described [19]. Plasmid pRL-TK constitutively expressing renilla luciferase was obtained from Promega. siRNAs specifically targeting hHerC6 (CUGUGAUGCAUGAUUCUAAtt), mISG15 (CCAUGACGGUGUCAGAACUUU) and mHerC6 (CACCAUACCUUAUACUGAAtt and GCAACUAUCGGUUGGAUUUtt), as well as a scrambled control were purchased from Ambion.

For the creation of human/mouse chimeric HerC6 expression constructs, unique KpnI and NotI sites were inserted upstream of the start codon and downstream of the stop codon, respectively, in both parental constructs using site directed mutagenesis (QuickChange XL, Stratagene). Subsequently, silent mutations were introduced to create unique XhoI sites in the middle of the ORFs between the RLD and HECT domain encoding sequences. Ultimately, chimeric constructs were generated by exchanging the KpnI-XhoI fragment or the XhoI-NotI fragment in the mHerC6 clone with the equivalent region of the hHerC6 clone. The resulting plasmids encode HA-tagged chimeric HerC6 proteins that are fused between the conserved amino acids L461 and E462.

### Transfection

Plasmid DNA transfections were performed in 6-well format with 1 µg of plasmid DNA at 30% confluence using either Fugene6 (Roche) for HEK-293T or Lipofectamine 2000 (Invitrogen) for HeLa and L-929. siRNA was transfected using Lipofectamine RNAiMAX (Invitrogen) in 12-well format. Transfections for immunofluorescence were carried out in suspension with 500 ng of plasmid DNA and 10^5^ cells in 12-well format. HeLa and L-929 cells were stimulated 16–24 h post transfection (hpt) with 1000 IU/mL (for RT-qPCR) or 10,000 IU/mL (for protein analysis) of universal IFN-β (PBL).

For reporter assays HEK-293T cells were seeded in 24-well clusters, to reach ∼30% confluence 16 hrs later at the time of transfection. Each well of the cluster was transfected with a total of 575 ng DNA. Each transfection contained 50 ng firefly luciferase reporter plasmid, 25 ng renilla luciferase reporter plasmid (pRL-TK) and 2 ng flag-RIGI(2CARD) plasmid. Each transfection mix was supplemented with 500 ng Herc5/6 plasmid. A plasmid expressing GST was included in each experiment as a base-line control. All samples were transfected in triplicate. Transfection complexes were formed with 2 ul Fugene6 (Roche) in 20 ul OptiMEM at room temperature for 30 minutes and directly added to complete growth medium on the cells.

Twenty-four hrs after transfection, cells were lysed in passive lysis buffer (Promega) and dual-luciferase activity was measured using the Dual-luciferase reporter assay system (Promega) in a Biotek Synergy 4 plate reader. Firefly luciferase values were normalized to renilla luciferase values. Fold increase of the firefly reporter was calculated relative to the GST baseline control in each experiment.

### ISGylation assays

Samples were lysed in disruption buffer (3 M Urea, 1 M β-mercaptoethanol and 2% SDS), boiled for 10 min. and analyzed for ISG15 conjugate formation by Western blot. HEK-293T cells were harvested after 24 h, whereas HeLa and L-929 cells were collected 48 h after IFN-β stimulation. Proteins were separated by SDS-PAGE on 4–15% gradient gels (Biorad) and subsequently transferred to an Immobilon-P membrane (Millipore) using a semi-dry transfer system (Biorad). Western blot analysis was performed using SNAP i.d. (Millipore) with either an HRP-conjugated V5 mAb (Serotec, MCA1360P), an anti-HA mouse mAb (Sigma, H9658) or a rabbit polyclonal anti-β-actin Ab (Sigma, A2103). Secondary HRP-conjugated sheep anti-mouse Ig and donkey anti-rabbit Ig Abs (GE Healthcare) were used for detection.

### Real-time RT-PCR

Total RNA was isolated from HeLa and L-929 cells 8 h after IFN-β stimulation using RNeasy columns (Qiagen) and treated for 30 min with Turbo DNase (Ambion). One µg of DNase-treated RNA was reverse transcribed using the iScript cDNA synthesis kit (Biorad). Real-time PCR was performed in 384 well plates in triplicate using gene specific primers for hHerC5 (fw_TCATTCTCCACCCCAAGAAG and rev_ACCTTTGCAAATGGAACCAC), hHerC6 (fw_TGACACAAGCAAGCCAACTC and rev_CATCCACGAAGTCTTCAGA) and mHerC6 (fw_TCCGGTGTTCTGAAACCTTC and rev_ CATCCTTCGCATTGAGGAAT) and Lightcycler480 SYBR green I mastermix (Roche) in a Roche LightCycler 480. Relative mRNA abundances were calculated using the ΔΔCt method [20] using 18S rRNA (fw_GTAACCCGTTGAACCCCATT and rev_CCATCCAATCGGTAGTAGCG) as a reference and plotted as fold change compared to mock-control samples.

### Immunofluorescence and confocal microscopy

HeLa cells were transfected in suspension as described above, seeded on glass coverslips, 24 h post transfection infected with Sendai virus for 8 hours and ultimately fixed in ice-cold absolute methanol. After blocking with 1% bovine serum albumin (BSA) in PBS, coverslips were incubated with primary antibodies diluted in 1% BSA in PBS for 1 h at room temperature (rt) and washed with PBS. Subsequently, coverslips were incubated with secondary antibodies as well as DAPI for nuclear staining (Invitrogen) for 30 min at rt and embedded in Prolong Gold (Invitrogen). Primary antibodies used are an anti-IRF3 mAb (Santa Cruz, FL-425, 1∶100), anti-HA mAb (Sigma, H9658, 1∶500) and polyclonal anti-Sendai serum (Charles River, 1∶400). Secondary Alexa Fluor 488-conjugated anti-mouse Ig and Alexa Fluor 633 anti-rabbit Ig Abs (Invitrogen) were used for detection. Confocal laser scanning was performed using a Zeiss LSM 510 Meta (Carl Zeiss) fitted with a Plan Apochormat 63×/1.4 oil objective. Images were collected at a resolution of 1024 by 1024 pixels and processed using Zeiss LSM Image Browser (Carl Zeiss).

## Supporting Information

Figure S1 **Knock-down of mHerC6 in mouse cells attenuates global ISGylation.** L-929 cells were transfected with a V5-tagged mouse ISG15 plasmid and indicated siRNAs, stimulated with IFN for 48 h and subsequently analyzed for global ISG15 conjugation by V5-specific immunoblot.
(TIF)
