# Supplementary figures and images for: HERC6 Is the Main E3 Ligase for Global ISG15 Conjugation in Mouse Cells
Source: PLoS One. 2012 Jan 17;7(1):e29870. doi: 10.1371/journal.pone.0029870 (PMC3260183; doi:10.1371/journal.pone.0029870)

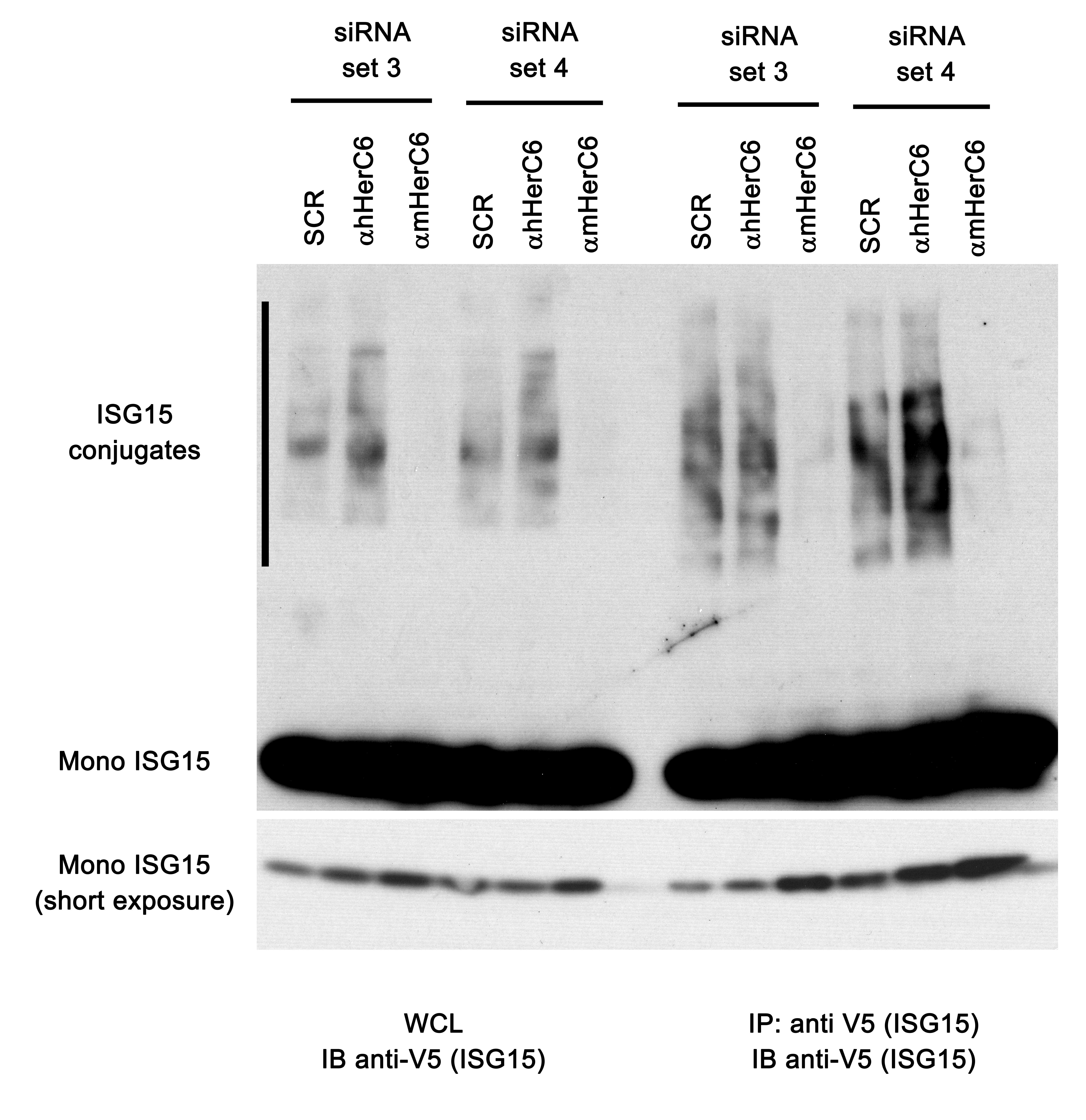

Supplement: Figure S1 — Knock-down of mHerC6 in mouse cells attenuates global ISGylation. L-929 cells were transfected with a V5-tagged mouse ISG15 plasmid and indicated siRNAs, stimulated with IFN for 48 h and subsequently analyzed for global ISG15 conjugation by V5-specific immunoblot. (TIF) [file pone.0029870.s001.tif]
